# Advancements in Nanoparticle-Based Adjuvants for Enhanced Tuberculosis Vaccination: A Review

**DOI:** 10.3390/vaccines12121335

**Published:** 2024-11-27

**Authors:** Jiao Wang, Zian Zhao, Quan Wang, Jingyu Shi, Duo Wai-Chi Wong, James Chung-Wai Cheung

**Affiliations:** 1 Department of Biomedical Engineering, Faculty of Engineering, The Hong Kong Polytechnic University, Hong Kong 999077, China; 2 Department of Clinical Laboratory, Hubei Provincial Hospital of Traditional Chinese Medicine, Wuhan 430073, China

**Keywords:** tuberculosis, vaccines, nanoparticles, adjuvants

## Abstract

Tuberculosis (TB) remains a leading cause of morbidity and mortality worldwide, necessitating the development of more effective vaccines. Nanoparticle-based adjuvants represent a promising approach to enhancing tuberculosis vaccine efficacy. This review focuses on the advantages of nanoparticulate-loaded vaccines, emphasizing their ability to improve antigen delivery, safety, and immunogenicity. We discuss the various types of nanoparticles and their unique physicochemical properties that contribute to improved antigen delivery and sustained immune activation. Additionally, we highlight the advantages of nanoparticle-based adjuvants in inducing strong cellular and humoral immunity, enhancing vaccine stability, and reducing adverse effects. Finally, we address current challenges and future perspectives in the application of these novel adjuvants, emphasizing their potential to transform TB vaccine strategies and ultimately contribute to better global health outcomes.

## 1. Introduction

Tuberculosis (TB), caused by the bacterium *Mycobacterium tuberculosis* (*M. tuberculosis*), remains a significant global health challenge, with an estimated 10 million new cases and 1.5 million deaths annually [1,2], as reported by the World Health Organization (WHO). This infectious disease primarily affects the lungs but can also manifest in other organs, leading to extrapulmonary TB. The burden of TB is particularly pronounced in low- and middle-income countries [3], particularly in Asia and Africa, where inadequate healthcare infrastructure, high rates of comorbidities like HIV [4], and the emergence of drug-resistant strains (multidrug-resistant TB, MDR-TB [5], and extensively drug-resistant TB, XDR-TB [6,7]) complicate control efforts. The only currently approved vaccine, Bacillus Calmette-Guérin (BCG) [8], while providing some protection against severe forms of TB in children [9], exhibits inconsistent efficacy in preventing adult pulmonary TB with highly variable protection ranging from 0% to 80%, which is highly variable and generally inadequate [10,11,12]. Furthermore, BCG fails to prevent reactivation of latent TB infections, which remains a major source of new cases.

To address these limitations, numerous new vaccine candidates are under development, targeting both pre-exposure and post-exposure scenarios. However, the absence of a highly effective TB vaccine underscores the need for innovative strategies that can enhance vaccine efficacy, safety, and stability. Nanoparticle-based adjuvants represent a promising advancement in the quest for more effective TB vaccines, offering several advantages over adjuvant candidates such as alum, MF59, and AS01 [13,14]. Unlike these conventional adjuvants, which may enhance only specific aspects of the immune response, nanoparticle formulations can be engineered for targeted delivery to antigen-presenting cells, facilitating improved antigen uptake and sustained release and ensuring prolonged exposure to the immune system [15,16]. This targeted approach not only enhances immunogenicity-promoting robust cellular and humoral responses but also allows for effective immune activation with lower doses of antigens, potentially reducing the overall amount needed and potentially decreasing the frequency of vaccinations required [17,18]. Their biocompatibility and safety profile are superior to traditional adjuvants. Furthermore, the modular design of nanoparticles enables the incorporation of multiple antigens or immunostimulatory agents within a single formulation, fostering a comprehensive immune response that is critical for combating TB [14,19]. Earlier research demonstrated that antigens incorporated into nanoparticulate systems can effectively amplify immunogenic responses against various pathogens responsible for infectious diseases. This suggests a similar promising application for tuberculosis vaccines once developed [20,21]. Nanoparticle-based adjuvants offer significant advantages over traditional vaccine formulations by improving antigen delivery, boosting immune responses, and reducing adverse effects [22,23,24,25]. All these features position nanoparticle-based adjuvants as a transformative strategy for enhancing TB vaccination efforts and addressing the ongoing global health challenge posed by this disease.

These innovative platforms facilitate targeted delivery to antigen-presenting cells (APCs), enhancing antigen uptake and presentation. Experimental studies have highlighted the potential of biocompatible and nontoxic methods in establishing prolonged immunity [22]. Moreover, incorporating adjuvants either triggers innate immunity or serves as a delivery mechanism to direct antigens specifically to APCs. This process promotes both cell-mediated and humoral immune responses through the activation of major histocompatibility complex (MHC) molecules [26]. The unique physicochemical properties of nanoparticles allow for controlled and sustained release of antigens, promoting long-lasting immune activation. Moreover, nanoparticles can be engineered to co-deliver multiple antigens or adjuvants, tailoring the immune response to effectively combat *M. tuberculosis* [15]. By protecting encapsulated antigens from degradation and improving bioavailability, nanoparticle-based adjuvants ensure that vaccines maintain their potency until reaching target tissues. In addition to enhancing immunogenicity, nanoparticle-based adjuvants can minimize adverse effects through targeted delivery, reducing off-target immune activation and lowering the required doses of antigens. This dual benefit not only improves safety but also optimizes the overall efficacy of TB vaccination strategies.

As research progresses, nanoparticle-based adjuvants hold transformative potential for TB vaccine development. By addressing the shortcomings of current vaccines and enabling new approaches for effective immune responses, these adjuvants could significantly impact global TB control efforts. Ultimately, the integration of nanoparticle-based adjuvants into TB vaccination strategies has the potential to contribute to better health outcomes worldwide, aligning with the WHO’s End TB Strategy and Sustainable Development Goals.

## 2. Types of Nanoparticles Used in TB Vaccines

**Figure 1:**
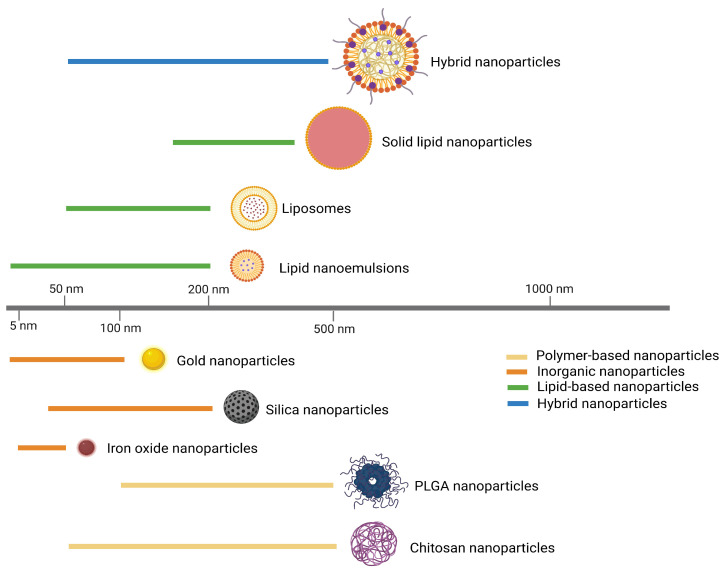
Average size range of different nanoadjuvants: lipid-based nanoparticles (50–500 nm); polymer-based nanoparticles (50–500 nm); inorganic nanoparticles (<200 nm); hybrid nanoparticles (50–500 nm).

In TB vaccine research, the size of nanoparticles used can vary depending on the type of material and the specific application. Generally, nanoparticles used as adjuvants in TB vaccines are within the range of 20 nm to 500 nm [16,27,28,29] (Figure 1). Due to their small size, nanoparticles exhibit unique physical, chemical, and biological properties that differ significantly from their bulk counterparts [15,30]. In the context of vaccinology, nanoparticles can be engineered from various materials, including lipids, polymers, metals, and proteins, to serve as carriers for antigens and adjuvants (Table 1).

### 2.1. Lipid-Based Nanoparticles

Lipid-based nanoparticles, such as liposomes, solid lipid nanoparticles (SLNs), and nanostructured lipid carriers (NLCs), are widely used in TB vaccine formulations due to their biocompatibility, ability to encapsulate both hydrophilic and hydrophobic antigens, and ease of surface modification. Liposomes are highly versatile and effective delivery systems, offering advantages such as biocompatibility, high loading capacity, and customizable surface modifications [31]. Composed of phospholipid bilayers, they mimic natural cell membranes, facilitating fusion with antigen-presenting cells (APCs) and enhancing antigen uptake. Additionally, liposome-based systems protect DNA from endonuclease degradation, significantly improving plasmid-DNA transfection and antigen presentation compared to naked DNA vaccines [32]. SLNs and NLCs offer advantages in terms of improved stability and controlled release, making them suitable for sustained antigen release and long-term immune stimulation [15]. Additionally, lipid-based nanoparticles can be engineered to target specific tissues or cells by conjugating targeting ligands, further enhancing their effectiveness as vaccine delivery platforms.

### 2.2. Polymer-Based Nanoparticles

Polymer-based nanoparticles, such as those made from poly (lactic-co-glycolic acid) (PLGA) [46,47], chitosan, and polyethylene glycol (PEG), are another class of nanocarriers used in TB vaccine development [48]. These nanoparticles can be engineered to tailor immune responses, making them suitable for TB vaccination. PLGA nanoparticles are highly valued for their biodegradability, FDA approval, and ability to provide controlled antigen release over extended periods, making them cost-effective and versatile for TB DNA vaccine development [49,50,51,52]. These nanoparticles protect encapsulated antigens and adjuvants from enzymatic degradation while enhancing immunogenicity. Chitosan-based nanoparticles, derived from natural polysaccharides, are ideal for mucosal TB vaccines due to their mucoadhesive properties, efficient antigen transport, and ability to open tight cell junctions, enabling effective nasal DNA vaccine delivery [53,54,55]. PEGylated nanoparticles can evade the mononuclear phagocyte system, prolonging circulation time and improving antigen presentation [56,57]. By adjusting the polymer composition and surface properties, polymer-based nanoparticles can be tailored to achieve desired immune responses and biodistribution profiles.

### 2.3. Inorganic Nanoparticles

Inorganic nanoparticles, including gold nanoparticles (AuNPs), silica nanoparticles, and metal oxides (e.g., iron oxide), are increasingly being explored for TB vaccine applications because of their unique physicochemical properties like ease of functionalization and stability. AuNPs, for example, are highly versatile and can be functionalized with various biomolecules to enhance antigen stability and uptake by dendritic cells (DCs). Several studies confirm the efficacy of AuNPs for DNA vaccination by injection via T cell activation and protection [41,58,59,60]. With their capacity for cargo delivery, biocompatibility, and ease of synthesis, AuNPs have been frequently studied as delivery vehicles [61]. Their inherent optical properties also allow for real-time tracking and monitoring of antigen delivery and immune response. Silica nanoparticles, with their high surface area and porous structure, enable efficient antigen loading and controlled release, while metal oxide nanoparticles can act as both delivery vehicles and imaging agents, aiding in the visualization of immune responses [18]. Inorganic nanoparticles can be designed to overcome delivery barriers and shape adaptive immunity, making them promising candidates for future TB vaccine development [23,62].

### 2.4. Hybrid Nanoparticles

Hybrid nanoparticles combine the properties of two or more materials, such as lipids, polymers, and inorganic components, to achieve synergistic effects. These nanoparticles can integrate the biocompatibility of lipid-based systems, the tunability of polymer-based carriers, and the structural stability of inorganic nanoparticles. For instance, lipid–polymer hybrid nanoparticles can provide the controlled release of polymer-based systems with the biocompatibility and cellular uptake efficiency of lipid-based carriers [19]. The use of lipid–polymer hybrid nanoparticles, such as those combining PLGA with DDA and TDB, has demonstrated the ability to tailor humoral and cellular immunity, which is crucial for protection against intracellular bacteria like *M. tuberculosis* [19]. Another study highlighted the use of biocompatible polymer poly (methyl methacrylate) (PMMA) in combination with cationic lipids to create hybrid nanoparticles that overcame the poor biocompatibility of traditional lipid-based adjuvants, suggesting their potential for a variety of vaccines, including TB [63]. Similarly, inorganic–lipid hybrids can enhance antigen stability and improve delivery efficiency. The versatility of hybrid nanoparticles allows for the incorporation of multiple functionalities, such as targeted delivery, enhanced antigen protection, and multimodal imaging, making them highly adaptable platforms for developing next-generation TB vaccines.

Each type of nanoparticle offers distinct advantages and can be tailored to address specific challenges in TB vaccine development (Table 2). By leveraging the unique properties of these diverse nanocarriers, researchers can design more effective and safer TB vaccines that elicit strong and durable immune responses, ultimately contributing to improved TB prevention and control.

## 3. Physicochemical Properties and Their Role in Antigen Delivery

### 3.1. Physicochemical Properties

The physicochemical properties of nanoparticles, such as size, shape, surface charge, hydrophobicity/hydrophilicity, and composition, play a crucial role in determining their behavior in biological systems and their effectiveness as vaccine delivery platforms. These properties influence the nanoparticles’ interactions with cells, their biodistribution, the efficiency of antigen delivery, and the type of immune response they elicit.

The size of nanoparticles significantly affects their uptake by APCs and subsequent trafficking within the body. The immunogenicity of smaller particles surpasses that of larger ones, making particle size a critical factor in using nanoparticles as vaccine adjuvants or delivery systems [72]. Nanoparticle-based tuberculosis vaccine delivery systems with sizes ranging from 300 to 600 nm are efficiently taken up by APCs and promote a Th1 immune response, essential for combating TB infection. In contrast, micron-sized particles tend to induce Th2 responses [68]. Nanoparticles with sizes ranging from 20 to 200 nm are generally considered ideal for vaccine delivery because they can efficiently be taken up by APCs such as DCs and macrophages [73]. Smaller nanoparticles (20–50 nm) can penetrate deeper into tissues and facilitate intracellular delivery, whereas larger nanoparticles (100–200 nm) are more suitable for lymph node targeting [16,74,75,76]. For instance, chitosan nanoparticles with an average diameter of 189 nm showed effective antigen delivery and sustained release, which is crucial for prolonged immune stimulation [77]. Optimal size selection ensures efficient cellular uptake and enhances antigen presentation to T cells, leading to robust immune activation.

The shape of nanoparticles influences their cellular uptake and distribution within the body. Spherical nanoparticles are more readily internalized by cells compared to rod-shaped or elongated particles, which can remain in circulation longer and exhibit different biodistribution patterns. Examples are the lipid nanoparticles (LNPs) which assume a micelle-like structure, encapsulating therapeutic molecules in a nonaqueous core [78]. Elongated or rod-shaped nanoparticles can enhance cellular interactions by increasing the contact area with the cell membrane, which may benefit specific antigen delivery strategies aimed at targeting tissues or organs involved in TB infection, such as lungs. Rod-shaped nanoparticles, such as aluminum oxyhydroxide nanorods, have been shown to induce higher levels of cellular uptake and immune activation compared to spherical particles [79]. Hydroxyapatite nanorods with a high aspect ratio were found to induce stronger pro-inflammatory responses and higher antigen-specific antibody production than their spherical counterparts [80].

The surface charge of nanoparticles determines their interaction with cell membranes, which are typically negatively charged. Positively charged nanoparticles exhibit higher affinity for cell membranes, leading to enhanced uptake by APCs [61,73,81]. PLGA nanoparticles modified with cationic surfactants and immunopotentiators were shown to induce strong Th1/Th17 responses, which are essential for protection against *M. tuberculosis* [19]. Comparative studies by Yan et al. demonstrated that among neutral, negatively, and positively charged liposomes, only cationic liposomes could upregulate the CCL2 chemokine gene in DCs. This induction was mediated through the ERK pathway both in vitro and in vivo [82]. Similarly, NH2-functionalized aluminum oxyhydroxide nanorods exhibited higher cellular uptake and NLRP3 inflammasome activation, leading to stronger immunogenicity [79]. The surface charge of adjuvants also influences the degree of association of antigens and the physical stability of vaccine formulations [73,83]. However, excessive positive charge can cause toxicity and lead to aggregation. Therefore, a balance must be achieved to maximize uptake without compromising safety. Neutral or slightly anionic nanoparticles can also facilitate longer circulation times, which may be beneficial for certain vaccine delivery applications [61,73].

The hydrophobic or hydrophilic nature of nanoparticles affects their stability in biological fluids, interactions with proteins, and cellular uptake. Hydrophobic nanoparticles may exhibit strong interactions with cell membranes, promoting uptake but potentially causing protein aggregation and rapid clearance from the bloodstream [73,83]. Hydrophobicity of microparticles is a key factor for determining the magnitude of immunogenicity stimulated by vaccination containing different particulate systems [84]. Increased surface hydrophobicity on polymeric microparticles, such as PLGA-based microparticles, was found to promote antigen internalization into DCs and enhance cytokine secretion levels, thereby boosting the immune response [84]. Hydrophilic nanoparticles, such as those coated with PEG, are more resistant to protein adsorption and can evade clearance by the mononuclear phagocyte system, resulting in prolonged circulation times [85,86]. Hydrophobic amino acid-based nanoparticles also demonstrated effective immune responses by leveraging their hydrophobic properties to enhance antigen delivery [87]. Modifying the surface hydrophobicity/hydrophilicity balance is key to optimizing nanoparticle behavior in vivo.

The composition of nanoparticles determines their biodegradability, biocompatibility, and ability to deliver antigens effectively. The chemical components used to prepare particulate vaccine adjuvants significantly influence their adjuvant potency [73]. Each nanoparticle material presents unique advantages, allowing for the design of optimized vaccine delivery systems.

### 3.2. The Role in Antigen Delivery

The surface of nanoparticles can be functionalized with various ligands, such as antibodies, peptides, or carbohydrates, to enhance targeting specificity. For TB vaccines, targeting ligands can be used to direct nanoparticles to specific receptors on APCs, such as mannose receptors, which are highly expressed on DCs [15]. For instance, guanidinylated cationic nanoparticles were shown to enhance antigen uptake and presentation by DCs, leading to a robust immune response [88]. Similarly, the use of Toll-like receptor (TLR) ligands as surface functionalizers can significantly boost the immunogenicity of the encapsulated antigens [89]. This targeted approach not only improves the efficiency of antigen delivery to immune cells but also reduces off-target effects and systemic distribution, minimizing potential side effects.

**Figure 2:**
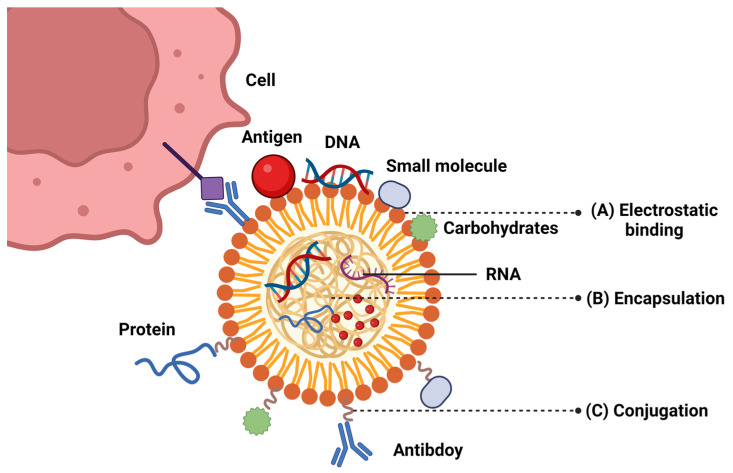
Schematic representation of nanocarriers. Different approaches can be utilized for the incorporation of targeting molecules (e.g., antigen, adjuvants, ligands, etc.) into/onto liposomes.

The ability of nanoparticles to encapsulate or conjugate antigens is another critical factor influencing their performance as vaccine carriers. As shown in Figure 2, nanoparticles offer high encapsulation efficiency, allowing for the presentation of multiple antigens directly to APCs while protecting them from early enzymatic and endosomal degradation, even under high-temperature conditions. This ensures that antigens, along with adjuvants, can reach the cytosol and initiate signaling cascades via TLRs, facilitating immediate immune stimulation, as highlighted in the literature [90]. The high encapsulation efficiency and antigen-loading capacity ensure that an adequate amount of antigen is delivered to target cells while preserving stability and enabling controlled release [15,91]. In one study, PLGA nanoparticles encapsulated with Angelica sinensis polysaccharide and ovalbumin showed an encapsulation efficiency of around 66.28%, which was effective in inducing a strong immune response [92]. PLGA nanoparticles have demonstrated high encapsulation efficiencies, making them suitable for vaccine delivery [92,93]. Nanoparticles with a high surface-to-volume ratio can encapsulate multiple antigens or adjuvants, providing opportunities for multivalent vaccine formulations. For instance, guanidinylated nanoparticles were able to load approximately 200 µg of ovalbumin per mg of polymer, which significantly enhanced the antigen-specific immune response [88]. Similarly, mesoporous silica nanoparticles (MSNs) have shown potential for high antigen loading, making them effective carriers for vaccine delivery [42]. Higher antigen loading can lead to a more robust immune response.

The degradation rate of nanoparticles and the release kinetics of the encapsulated antigen are crucial determinants of the duration of immune stimulation. Nanoparticles can be engineered to degrade at specific rates, ensuring a sustained release of the antigen. Nanoparticles made from biodegradable polymers like PLGA can provide sustained antigen release over days or weeks, ensuring prolonged immune activation and memory formation [35,94,95]. Similarly, silk fibroin nanoparticles demonstrated a continuous and slow release of the antigen over 56 days, which helped in maintaining a prolonged immune response [96]. By adjusting the polymer composition or incorporating additional stabilizing agents, it is possible to fine-tune the degradation and release profiles to match the desired immune response.

By carefully engineering these physicochemical properties, researchers can optimize nanoparticles to enhance antigen stability, improve cellular uptake, promote targeted delivery, and elicit desired immune responses. This rational design approach holds promise for developing next-generation TB vaccines that are safer, more effective, and capable of providing long-lasting protection against the disease.

## 4. Mechanisms of Improved Antigen Delivery and Immunogenicity of Nanoparticle-Based TB Vaccines

Currently, the majority of adjuvants effective in TB animal models, including those in clinical development, share a common feature: they combine the dual functionality of an immunomodulator, which activates pathogen-recognition receptors (PRRs), and a delivery system that ensures efficient vaccine targeting [97,98]. Nanoparticle-based adjuvants offer unique mechanisms that enhance the delivery and immunogenicity of TB vaccines, overcoming several limitations of traditional vaccine strategies. These mechanisms include targeted delivery to APCs, controlled and sustained antigen release, improved cross-presentation, and the ability to serve as multifunctional platforms for the co-delivery of antigens and additional adjuvants.

### 4.1. Targeted Delivery and Uptake by Antigen-Presenting Cells

Nanoparticles offer unique capabilities to enhance antigen delivery specifically to APCs, such as DCs and macrophages, thereby optimizing immune activation and antigen presentation. Antigen presentation by APCs is a crucial step that links innate and adaptive immunity and involves distinctive mechanisms [4].

Nanoparticles can be functionalized with surface ligands (e.g., mannose, antibodies, or peptides) that specifically bind to receptors on APCs, such as C-type lectin receptors, scavenger receptors, or DC-SIGN [99,100]. These modifications increase the affinity and specificity of nanoparticles for APCs, ensuring that the encapsulated antigen is preferentially delivered to these immune cells rather than being distributed nonspecifically throughout the body. For example, liposomes modified with appropriate targeting ligands activate cells via pattern-recognition receptors, leading to the maturation of APCs and efficient antigen processing and presentation [15]. Surface modifications of PLGA nanoparticles with biomaterials like mannose or cationic lipids, or the use of adjuvants, have improved antigen presentation to DCs, although this may come at the cost of reduced encapsulation efficiency, morphology, and yield [45,101].

The physicochemical properties of nanoparticles play a critical role in determining their interaction with APCs. Nanoparticles typically range from 20 to 200 nm in size, a range optimal for endocytosis and phagocytosis by APCs. Additionally, positively charged nanoparticles show enhanced uptake by APCs due to electrostatic interactions with the negatively charged cell membranes. For instance, the positively charged surface of cationic liposomes favors interactions with the negatively charged surface of DCs, facilitating antigen delivery and uptake [102]. These nanoparticles stimulate the maturation of DCs and the activation of macrophages, leading to a higher production of cytokines such as TNF-α, IL-6, and IL-10, which are essential for a strong immune response [88]. Additionally, cationic lipid nanoparticles have been formulated with triple adjuvants to enhance immune responses against respiratory pathogens, suggesting their potential for TB vaccination [103]. The surface charge and particle size of liposomes influence vaccine delivery by enhancing their ability to attract, interact with, and activate APCs, such as DCs, macrophages, and B cells [104]. Shape also influences uptake, as rod-shaped or discoidal nanoparticles have shown superior internalization compared to spherical particles in some studies.

Once inside APCs, nanoparticles can facilitate efficient antigen processing and presentation via both MHC class I and class II pathways. This dual presentation is crucial for the activation of both CD8^+^ cytotoxic T cells and CD4^+^ helper T cells, leading to a more comprehensive immune response [15,105,106]. Additionally, some nanoparticles can promote the escape of antigens from endosomes into the cytosol, further enhancing cross-presentation and the generation of a robust cellular response. Another study demonstrated that lipid nanoparticles could significantly enhance B cell and T cell responses to subunit vaccine antigens, indicating their potential as adjuvants in TB vaccines [107]. LNPs also enhance the uptake of antigens by APCs, leading to stronger CD4^+^ and CD8^+^ T cell responses [107].

By targeting delivery specifically to APCs, nanoparticles prevent premature degradation of the antigen in the extracellular environment. This targeted approach ensures that a higher proportion of the administered antigen reaches its intended cellular target, leading to a more effective and efficient immune activation. For instance, guanidinylated cationic nanoparticles were shown to encapsulate antigens effectively and release them in a controlled manner within the acidic lysosomal compartments of APCs. This targeted delivery not only protects the antigen from degradation but also enhances its uptake and presentation by APCs, leading to a more robust immune response [88]. Lipid-based nanoparticles were demonstrated to enhance the delivery of antigens to lymph nodes, where they are efficiently taken up by APCs [15].

### 4.2. Controlled and Sustained Release of Antigens

Nanoparticles can be engineered to control the release kinetics of encapsulated antigens, providing sustained exposure to the immune system over time. This prolonged release enhances the magnitude and duration of the immune response, which is critical for effective TB vaccination.

Encapsulation in nanoparticles protects antigens from harsh environmental conditions, such as low pH or enzymatic degradation, during storage or after administration. Polymers like PLGA and chitosan are commonly used to encapsulate antigens in nanoparticles. These polymers degrade slowly over time, releasing the encapsulated antigen in a controlled manner. This gradual degradation ensures a steady supply of antigen to APCs, mimicking the natural pathogen persistence and resulting in prolonged immune stimulation. PLGA possesses immunological properties, such as receptor recognition through cytokine stimulation and APCs, which protect vaccines from enzymatic degradation and offer greater immune safety compared to live bacterial carriers [108,109]. The use of lipid-based nanoparticles also allows for the encapsulation of antigens and adjuvants, providing a controlled release that enhances both humoral and cellular immune responses [15]. This protection ensures that the antigen remains in its native conformation, preserving its immunogenicity and enhancing its recognition by the immune system.

The release rate of antigens can be fine-tuned by altering the properties of the nanoparticles, such as polymer composition, molecular weight, and hydrophobicity. For example, increasing the hydrophobicity of the polymer matrix or cross-linking density can slow down the release rate, leading to prolonged antigen availability. MnO_2_ nanoparticles not only deliver antigens but also act as a depot to release Mn^2+^ ions, which enhance the immune response through the stimulator of interferon genes (STING) pathway in DCs [110]. This feature is particularly important for generating memory responses, as it allows for continuous stimulation of the immune system without the need for multiple booster doses.

By providing a steady release of antigens, nanoparticles ensure sustained activation of APCs, which, in turn, continue to present antigens to T and B cells over an extended period. Particle replication in nonwetting template (PRINT) nanoparticles were shown to sustain prolonged antigen presentation to APCs, leading to a stronger and more sustained immune response compared to rapidly clearing soluble antigens [111]. This prolonged presentation increases the likelihood of forming long-lived memory T cells and effector T cells, leading to long-term protection against TB.

### 4.3. Multivalent and Multifunctional Nanoparticles

Nanoparticles can be designed to incorporate multiple antigens, adjuvants, or immunomodulatory molecules, thereby acting as multifunctional platforms capable of orchestrating complex immune responses [15,23,112]. This versatility enables nanoparticles to enhance the breadth and quality of the immune response.

Nanoparticles can encapsulate or conjugate multiple antigens that represent different stages or proteins of *M*. *tuberculosis* [45]. This multivalent approach ensures that the immune system is trained to recognize various targets on the pathogen, reducing the likelihood of immune escape and increasing vaccine efficacy [15]. For example, combining early secreted antigens (e.g., ESAT-6) with later-stage antigens (e.g., Ag85 complex) can generate a more comprehensive response capable of targeting both acute and latent TB infections [30].

Nanoparticles can be co-loaded with potent adjuvants, such as TLR agonists, cytokines, or small molecule immunomodulators, to further enhance immune responses [110,113]. For instance, lipid nanoparticles combined with a synthetic TLR9 agonist were shown to elicit stronger Th1-type responses and multifunctional CD8^+^ T cell responses compared to unadjuvanted vaccines [107]. These adjuvants can activate innate immune pathways, promote APC maturation, and induce the production of pro-inflammatory cytokines, thereby boosting the overall immune response to the antigen. Co-delivery of antigens and adjuvants within the same nanoparticle ensures that these signals are presented simultaneously to APCs, leading to synergistic effects and optimal immune activation [114,115]. Self-immolative micelles can co-deliver antigens and adjuvants to DCs, leading to the release of pristine antigens and the upregulation of costimulatory molecules, which are essential for a strong cellular immune response [116].

The surface of nanoparticles can be modified with pathogen-associated molecular patterns (PAMPs) or other targeting moieties to mimic natural pathogen structures. PAMPs are critical components in the design of nanoparticle-based adjuvants due to their ability to activate pattern-recognition receptors on immune cells [117,118]. The incorporation of multiple PAMPs into nanoparticles can mimic the natural infection process, leading to a more potent immune response [119,120]. This functionalization enhances the ability of nanoparticles to activate innate immune receptors, such as TLRs or NOD-like receptors (NLRs), resulting in stronger and more rapid immune responses. For instance, incorporating lipopolysaccharide or CpG motifs on the nanoparticle surface can activate TLR4 and TLR9 pathways [121,122,123], respectively, promoting a Th1-biased response crucial for TB protection. Similarly, nanoparticles co-loaded with CpG oligodeoxynucleotide (a TLR9 agonist) and 3pRNA (a RIG-I agonist) were shown to significantly enhance APC activation and antigen cross-presentation, resulting in strong humoral and cytotoxic T lymphocyte responses [115].

By delivering a combination of antigens and adjuvants that activate different immune pathways, multivalent nanoparticles can elicit both Th1 and Th17 responses, which are essential for protection against TB. Th1 responses promote IFN-γ production and macrophage activation, while Th17 responses enhance mucosal immunity and neutrophil recruitment [16,19,124]. Aluminum hydroxide nanoparticles co-loaded with CpG oligodeoxynucleotide (CpG-ODN) and 3pRNA activate TLR9 and retinoic acid-inducible gene I (RIG-I), respectively. This dual activation leads to robust humoral and cellular immune responses, including strong cytotoxic T lymphocyte (CTL) responses and IFN-γ secretion, which are crucial for effective TB vaccination [115]. This balanced activation is necessary for clearing both intracellular and extracellular *M. tuberculosis* bacilli.

**Figure 3:**
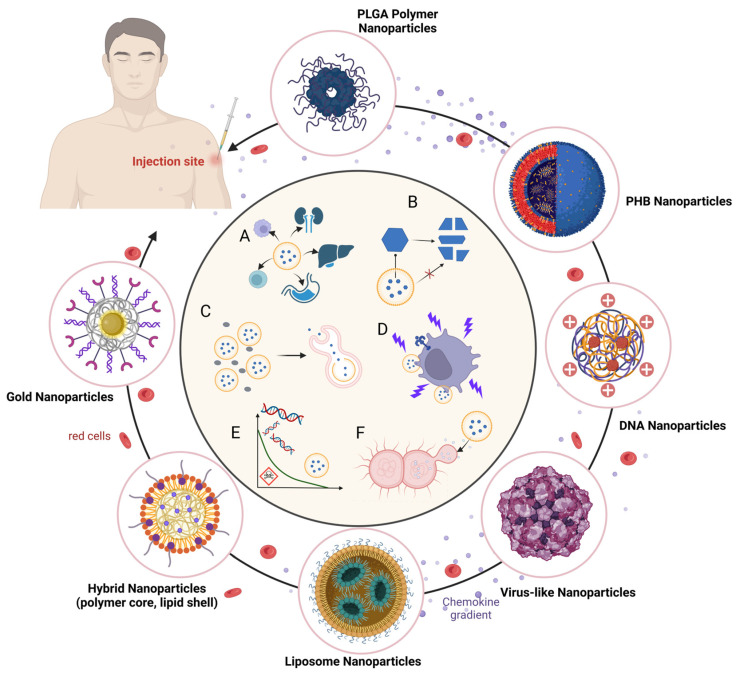
Characteristics of vaccine delivery systems with different nanocarriers in TB vaccines. A: target antigen to specific cells/organs. B: protect the antigen from degradation. C: slow release of antigen. D: enhance antigen uptake/presentation. E: dampen PAMP systemic toxicity. F: direct stimulatory effect of the APC or adjacent cells.

The ability to deliver multiple components in a controlled manner and target specific immune pathways makes nanoparticle-based TB vaccines a versatile and powerful tool for inducing robust and long-lasting immunity against *M. tuberculosis*. Figure 3 shows these features, positioning them as promising candidates to enhance current TB vaccination strategies and improve global health outcomes.

## 5. Advantages of Nanoparticle-Based Adjuvants in TB Vaccination

Nanoparticle-based adjuvants in TB vaccines enhance immune protection by inducing strong cellular and humoral responses, which are crucial for effective, long-term immunity against tuberculosis. They also improve the stability of vaccines and protect antigens from degradation, ensuring efficient delivery to target cells. Additionally, these adjuvants allow targeted delivery and controlled release, which helps minimize adverse effects and enables reduced antigen doses without sacrificing effectiveness. This combination of benefits makes nanoparticle-based adjuvants a promising approach for advancing TB vaccination strategies.

### 5.1. Induction of Strong Cellular and Humoral Immunity

**Figure 4:**
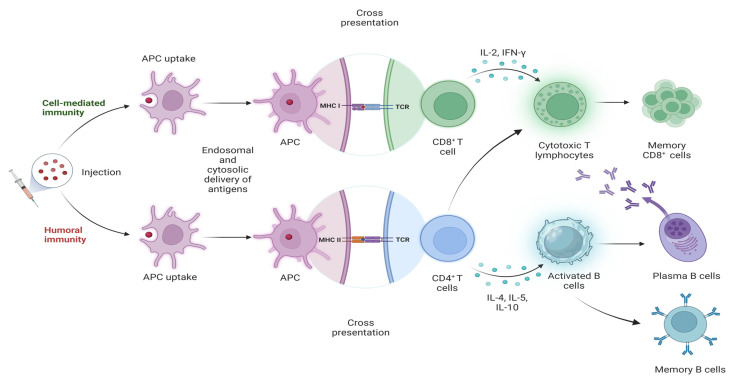
Different nanoparticle-based TB vaccine delivery systems function by activating cellular and humoral immunity to varying degrees.

Nanoparticle-based adjuvants have the capability to simultaneously stimulate robust T cell-mediated (Th1/Th17) and B cell-mediated responses, which are crucial for effective protection against *M*. *tuberculosis*. Unlike traditional adjuvants, which may primarily induce one type of immune response, nanoparticles provide a more balanced and comprehensive activation of both cellular and humoral immunity (Figure 4).

Nanoparticles enhance antigen uptake and processing by APCs, leading to the efficient presentation of antigens via both MHC class I and II pathways [125,126,127]. This dual pathway activation is essential for the differentiation of CD4^+^ T helper cells into Th1 and Th17 subsets and the activation of CD8^+^ cytotoxic T cells. Th1 cells produce cytokines such as IFN-γ and TNF-α, which activate macrophages to kill intracellular *M*. *tuberculosis*, while Th17 cells secrete IL-17, promoting mucosal immunity and recruitment of neutrophils to the site of infection [15]. Polypropylene sulfide nanoparticles conjugated with antigens were shown to enhance both MHC I and MHC II presentation pathways, leading to robust activation of both CD8^+^ and CD4^+^ T cells [126,128]. This comprehensive activation of T cells provides enhanced control over both acute and latent TB infections. Previous studies showed that TB vaccines encapsulating plasmid DNA and immunostimulants in PLGA nanoparticles induce higher levels of interferon-gamma (IFN-γ) and immunoglobulin (Ig) G2a antibodies in mice via injection compared to naked plasmid-DNA vaccines, indicating a strong Th1 polarization-directed response [129,130]. Furthermore, chitosan nanoparticles co-administered with mucosal adjuvants were found to stimulate strong Th1/Th17 responses and induce CD8^+^ T cells, which are essential for robust cellular immunity [131].

Although the role of humoral immunity in TB protection is less prominent compared to cellular immunity, antibodies can neutralize extracellular bacilli and prevent their dissemination. Nanoparticles enhance B cell activation by facilitating the delivery of antigens to follicular dendritic cells in lymphoid organs, promoting the formation of germinal centers where B cell maturation occurs [132,133]. This process leads to the generation of high-affinity antibodies and long-lived plasma cells, contributing to a strong and sustained humoral response [134]. The mechanisms by which nanoparticles enhance B cell responses and antibody production are multifaceted. One key mechanism is the ability of nanoparticles to target lymph nodes, where B cells are activated. For instance, PEGylated lipid nanoparticles encapsulating cyclic di-GMP (cdGMP) were shown to redirect the adjuvant to draining lymph nodes (dLNs), leading to increased germinal center B cell differentiation and higher antibody titers [113]. This targeted delivery ensures that the antigens and adjuvants are presented in an optimal environment for B cell activation and differentiation. Additionally, nanoparticles can modulate the immune response by activating specific pathways.

By providing prolonged antigen exposure and enhanced stimulation of APCs, nanoparticle-based adjuvants promote the formation of memory T and B cells [17,95,135]. These memory cells are crucial for long-term protection and rapid immune responses upon re-exposure to the pathogen, ensuring lasting immunity against TB. Nanoparticles can be engineered to target lymph nodes, where immune responses are initiated and memory cells are formed. For example, PEGylated lipid nanoparticles encapsulating cyclic dinucleotides (CDNs) were shown to enhance the accumulation of adjuvants in draining lymph nodes, leading to increased CD8^+^ T cell responses and durable antibody titers [113]. Certain nanoparticle formulations can induce tissue-resident memory T cells (TRM), which provide long-term protection at mucosal and barrier sites such as the lungs [136].

### 5.2. Enhanced Vaccine Stability and Antigen Protection

Nanoparticles offer significant advantages in terms of antigen stability and protection, ensuring that the vaccine remains potent and effective under various environmental conditions.

Encapsulation of antigens within nanoparticles shields them from enzymatic degradation by proteases and other enzymes present in the body. This protection is particularly important for peptide or protein antigens that are susceptible to rapid breakdown [114,137,138]. By preserving the integrity and conformation of the antigen, nanoparticles ensure that the antigen remains bioactive and capable of eliciting a strong immune response [18]. The encapsulation of antigens in PLGA nanoparticles was shown to increase the in vivo stability and lymph node accumulation of the antigenic peptide, thereby enhancing the overall efficacy of the vaccine [137]. Additionally, encapsulation within nanoparticles prevents antigen aggregation and the formation of inactive multimers, which can occur during storage or under harsh conditions [138]. This reduction in aggregation ensures that the antigen maintains its structural and functional properties, leading to a more consistent and effective immune response.

Nanoparticle-based formulations are more stable under varying pH and temperature conditions compared to traditional adjuvants or free antigen formulations [136,139]. This stability reduces the risk of antigen denaturation during storage and transport, making nanoparticle-based vaccines more suitable for use in diverse geographical regions, including areas with limited cold chain infrastructure [138,140]. One of the significant benefits of nanoparticle-based TB vaccines is their enhanced thermostability. For instance, spray drying has been used to encapsulate a TB subunit vaccine containing a nanoemulsion adjuvant into dry powder microparticles. This method has shown that the powder form of the vaccine remains physically stable for up to 26 months at temperatures as high as 40 °C. The integrity of the adjuvant system is maintained at temperatures up to 25 °C for 26 months and even after one month at 40 °C, demonstrating improved antigen thermostability compared to liquid formulations [138]. The use of cationic solid lipid nanoparticles (cSLNs) was demonstrated to enhance the stability of antigens, as evidenced by an increase in the transition temperature related to antigen dissociation, indicating improved stability [141].

Nanoparticles can be engineered to release their antigen payloads in a controlled and sustained manner, providing continuous stimulation to the immune system. This sustained release mimics the natural course of infection, where the immune system is exposed to antigens over a prolonged period. The result is a more effective induction of both primary and memory immune responses, which are essential for long-term protection against TB [103,112,142]. Additionally, targeted delivery to specific tissues or cells can reduce the exposure of the vaccine components to degrading environmental factors. This approach has been demonstrated in various studies, including the use of PLGA nanoparticles and lipid-based nanoparticles for TB vaccination [16,19].

### 5.3. Reduction of Adverse Effects

One of the key challenges in vaccine development is minimizing adverse effects while maintaining strong immunogenicity. By targeting antigens specifically to APCs or other immune cells, nanoparticles minimize off-target effects and reduce systemic exposure [17]. This targeted approach reduces the likelihood of nonspecific immune activation and systemic inflammatory responses, which are common with traditional adjuvants. Nanoparticle-based TB vaccines offer the advantage of precise targeting, which significantly reduces systemic distribution and associated adverse effects. Encapsulating adjuvants like cyclic dinucleotides (CDNs) within PEGylated lipid nanoparticles redirects the adjuvant to draining lymph nodes (dLNs) rather than the bloodstream, thereby minimizing systemic inflammation [113]. Similarly, synthetic vaccine particles (SVPs) co-encapsulating antigens and adjuvants result in strong local immune activation without inducing systemic cytokine release, thus reducing potential adverse events [143].

Nanoparticles enhance the potency of encapsulated antigens and adjuvants, allowing for lower doses to achieve the desired immune response. The use of PLGA:DDA hybrid nanoparticles for nasal delivery of TB antigens demonstrated efficient immune responses with lower doses, which can reduce the risk of adverse effects associated with higher doses [45]. This dose-sparing effect reduces the risk of local reactogenicity (e.g., pain, redness, or swelling at the injection site) and systemic side effects (e.g., fever, fatigue) associated with high-dose vaccinations.

Traditional adjuvants such as alum or oil-in-water emulsions can cause strong local inflammatory reactions. Nanoparticles, on the other hand, can be engineered to minimize local reactogenicity by modulating their surface properties, composition, and release kinetics. The co-delivery of antigens with adjuvants in synthetic polymer nanoparticles was shown to result in minimal systemic production of inflammatory cytokines, thereby reducing potential adverse events and safety concerns [143]. Using biocompatible and biodegradable materials like PLGA or chitosan can reduce local irritation and enhance biocompatibility, making nanoparticle-based vaccines more tolerable. The use of biocompatible materials such as chitosan for nanoparticle formulation ensures that the vaccine is nontoxic and well tolerated, further minimizing adverse effects [131].

The ability of nanoparticles to co-deliver antigens and immune-stimulatory molecules in a controlled manner prevents excessive immune activation, which can lead to adverse effects such as cytokine storms or autoimmune reactions. By carefully tuning the composition and release profile of nanoparticles, it is possible to achieve a balanced immune activation that is both potent and safe. The use of cationic lipid nanoparticles with a triple adjuvant system for intranasal vaccination against respiratory pathogens was shown to favor a Th1 type of response, promoting higher IgG2a and IgA serum antibody titers without causing excessive immune activation [103]. Additionally, the modular self-assembling nanoparticle platform used in SVPs enables strong local immune activation and balanced humoral and cellular responses without systemic overstimulation [143].

These advantages make nanoparticle-based adjuvants a promising platform for developing safer and more effective TB vaccines, addressing the limitations of traditional adjuvants and enhancing the overall efficacy and safety profile of TB vaccination strategies.

## 6. Current Technical and Manufacturing Challenges in the Development of Nanoparticle-Based TB Vaccines

Developing nanoparticle-based TB vaccines at a large scale presents numerous technical and manufacturing challenges that must be addressed to ensure the consistency and reproducibility of formulations [144]. While many nanoparticle formulations have shown promise at the laboratory scale, translating these methods to industrial-scale production is complex. This complexity arises due to the need for precise control over particle size, shape, surface characteristics, and encapsulation efficiency [51,145]. Small changes in manufacturing parameters can result in variations in these properties, which can significantly impact vaccine performance and safety [30]. Scaling up the production while maintaining these critical quality attributes requires sophisticated equipment and process optimization.

Consistency between different batches is crucial for regulatory approval and clinical application. Even minor variations in raw materials, environmental conditions, or processing steps can lead to inconsistencies in nanoparticle size, surface charge, or antigen loading, affecting the vaccine’s immunogenicity and safety [146,147]. Developing robust manufacturing protocols and in-process control measures is essential to ensure that every batch of nanoparticle-based vaccines meets stringent quality requirements. For instance, the engineering of lipid–polymer hybrid nanoparticles requires precise control over several critical process parameters (CPPs) such as acetone concentration, stabilizer concentration, and lipid-to-total solid ratio to achieve consistent particle size, polydispersity index (PDI), and zeta potential [19]. Variations in these parameters can significantly affect the immunogenicity and stability of the vaccine.

Developing multifunctional nanoparticles that can co-deliver multiple antigens and adjuvants in a controlled manner increases the complexity of the formulation. These delivery systems often require multistep processes, sophisticated techniques (e.g., emulsion–evaporation or microfluidic methods), and strict environmental controls, all of which add to the cost and difficulty of large-scale production [51]. For example, the development of mesoporous silica nanoparticles as vaccine carriers involves optimizing synthesis, adsorption, and release kinetics to achieve effective antigen delivery and immune response [42]. Nanoparticle-based formulations are often sensitive to environmental conditions such as temperature and humidity. This sensitivity can lead to issues related to the long-term stability of the vaccine, complicating storage and distribution, particularly in resource-limited settings [34,148]. Formulating nanoparticles to enhance their stability under a wide range of conditions or developing appropriate stabilization techniques (e.g., lyophilization) is necessary to ensure that the vaccines remain effective throughout their shelf life.

## 7. Future Perspectives and Transformative Potential

Nanoparticle-based adjuvants have the potential to transform TB vaccination by enhancing both efficacy and safety, allowing precise antigen delivery and minimizing side effects. Some nanoparticle-based TB vaccines, including RUTI and ID93 + GLA-SE, have entered preclinical and clinical trials [149,150,151,152,153], exhibiting favorable safety profiles and encouraging immunogenicity results (Table 3). These adjuvants also pave the way for effective mucosal TB vaccines, which target the respiratory tract directly, providing localized immunity at the primary infection site. Furthermore, integrating nanoparticle-based adjuvants into global TB control strategies could enhance vaccine accessibility and impact, supporting worldwide TB eradication efforts with a focus on long-lasting, robust immunity.

### 7.1. Innovative Approaches for Enhancing Efficacy and Safety

The future perspectives for nanoparticle-based TB vaccines are highly promising due to ongoing advancements in nanotechnology and vaccine design. Innovations in precision medicine enable the development of personalized nanoparticle vaccines, which can be tailored to an individual’s immune profile for a more effective response [158]. Smart-release systems using nanoparticles can be designed to respond to environmental cues like pH, allowing for precise antigen delivery and timing [159,160]. Moreover, emerging nanoparticle platforms can co-deliver multiple antigens or combine them with adjuvants, leading to a more robust immune response [161,162]. Hybrid nanoparticles and biomimetic approaches offer improved antigen delivery by mimicking natural pathogens and overcoming TB’s complex life cycle [19]. These advancements have the potential to transform TB vaccine strategies, enhancing both their efficacy and safety.

### 7.2. Potential for Mucosal TB Vaccines

Mucosal vaccination, particularly targeting the respiratory tract where *M. tuberculosis* initially infects, is a promising area for nanoparticle-based TB vaccine development [163]. The nasal cavity offers several advantages, including better patient compliance, improved epithelium permeability, and the potential for needle-free self-administration [164,165]. While mucosal vaccination holds significant promise for providing physiological and immunological protection against *M. tuberculosis*, safety concerns related to adjuvants and administration routes must be considered [70,166]. Evidence suggests that mucosal vaccination via the respiratory tract offers superior immune protection against pathogenic bacteria compared to other administration sites [167,168]. Additionally, PLGA/PLA nanoparticles administered intranasally or intratracheally have shown effectiveness in mediating protective immune responses against respiratory bacterial infections [169,170].

Mucosal administration of nanoparticle-based vaccines (e.g., intranasal or aerosol delivery) can induce strong local immunity at the primary site of TB infection [171]. Localized immunity at the respiratory mucosa is crucial for preventing the establishment of infection and controlling bacterial replication early in the infection process. Amini and colleagues developed a trimethyl chitosan (TMC)-based vaccine candidate for intranasal administration. These TMC nanoparticles encapsulate the antigen and release it gradually, enhancing uptake by immune cells in the nasal-associated lymphoid tissue and triggering a targeted immune response in the respiratory mucosa [172].

Mucosal vaccines are generally noninvasive and can be self-administered, making them more acceptable to patients and easier to deploy in large-scale vaccination campaigns. Mucosal nanoparticle-based vaccines can be used as booster vaccines in individuals previously vaccinated with BCG or other TB vaccines [18,173,174]. This strategy can enhance local immunity and provide an additional layer of protection without the need for multiple systemic doses.

### 7.3. Integration with Global TB Control Strategies

Nanoparticle-based TB vaccines hold significant promise in advancing global TB control efforts, aligning with the WHO’s End TB Strategy and Sustainable Development Goals (SDGs). By offering improved protection and reduced adverse effects and targeting latent TB infections, these vaccines could play a crucial role in achieving the End TB Strategy’s targets of reducing TB incidence by 90% and TB deaths by 95% by 2035 [175]. Additionally, South African researchers have advanced a nanoparticle-based vaccine formulation to clinical trials, utilizing a synthetic nanoemulsion adjuvant, GLA-SE, combined with the TB antigen ID93 [176]. The development of nanoparticle-based TB vaccines provides opportunities for global collaboration in research and development. Such collaborative efforts can accelerate the progress of TB vaccine research, foster innovation, and encourage the sharing of knowledge across borders.

## 8. Conclusions

In summary, nanoparticle-based adjuvants represent a promising advancement in TB vaccine development by offering improved antigen delivery, enhanced immune activation, and sustained antigen release, leading to potent cellular and humoral responses crucial for TB protection. Despite challenges such as manufacturing scalability, regulatory concerns, and accessibility in low-resource settings, these innovative platforms hold transformative potential. With further research and strategic implementation, nanoparticle-based vaccines could significantly contribute to global TB control efforts, especially through novel approaches like mucosal vaccination, ultimately supporting the WHO’s goal of eradicating TB.

## Figures and Tables

**Table 1:** Structural features and functions of nanoparticles as adjuvants in TB vaccines.

Category	Type	Structure	Function
Lipid-based	Liposomes [31,32]	- Phospholipid bilayer - Encapsulating hydrophilic antigens - Hydrophobic antigens	- Delivers TB antigens like Ag85B, ESAT-6 - Enhances antigen uptake and presentation
	SLNs [33]	- Solid lipid core w/ surfactant coating	- Enhances stability and immune response of TB antigens like Ag85A
	Lipid NEM [34]	- Oil-in-water/water-in-oil droplets - Stabilized by surfactants	- Delivers hydrophobic TB antigens - Promotes antigen uptake
Polymer-based	PLGA NPs [35,36]	- Biodegradable PLGA matrix - Encapsulating antigens	- Sustained release of antigens like HspX, ESAT-6 - Enhances immune response
	Chitosan NPs [37,38]	- Cationic chitosan structure - Interacting w/ negatively charged antigens	- Ideal for mucosal TB vaccine delivery - Enhances local immune responses
Inorganic	AuNPs [39,40,41]	- Gold core w/ tunable surface functionalization	- Delivers Mtb antigens like CFP-10 - Facilitates uptake by dendritic cells
	Silica NPs [42,43]	- Porous structure w/ high surface area	- Co-delivers antigens and adjuvants - Enhances stability and immune activation
	Iron oxide NPs [18,44]	- Magnetic core w/ modifiable surface	- Magnetic targeting of TB antigens - Imaging and diagnostics
Hybrid	Hybrid NPs [45]	- Combination of materials (e.g., lipid–polymer) - Multilayered	- Co-delivers antigens and adjuvants - Enhances stability and immune response

AuNPs: gold NPs; NEM: nanoemulsions; NPs: nanoparticles; PLGA: polylactic-co-glycolic acid; SLNs: solid lipid NPs; TB: tuberculosis; Mtb: Mycobacterium tuberculosis; w/: with.

**Table 2:** Advantages and disadvantages of nanoparticles as adjuvants in TB vaccines.

Category	Type	Advantages	Disadvantages
Lipid-based	Liposomes [32]	- Protects antigens - Increases antigen uptake; - Can be surface-modified for targeted delivery.	- Stability and leakage issues; - Scale-up difficulties.
	SLNs [33,64]	- Biocompatible and biodegradable; - Sustained release; - Good protection of antigens.	- Limited drug-loading capacity; - Lipid crystallization can affect release.
	Lipid NEM [65]	- High surface area; - Enhanced antigen stability; - Modifiable with immune-stimulatory molecules.	- Stability issues like coalescence; - Maintaining homogeneity.
Polymer-based	PLGA NPs [66,67]	- FDA-approved; - Sustained release; - Protection from degradation.	- Burst release; - Acidic degradation products affecting stability.
	Chitosan NPs [68]	- Good mucoadhesive properties; - Enhances antigen uptake; - Biodegradable.	- Limited solubility in physiological conditions; - Variability in properties.
Inorganic	AuNPs [39,40]	- Easy surface modification; - Biocompatible; - Useful for imaging and diagnostics.	- Long-term accumulation; - High production costs.
	Silica NPs [42,69]	- High surface area; - Good stability; - Customizable properties.	- Limited biodegradability; - Potential toxicity.
	Iron oxide NPs [18,44]	- Precise magnetic targeting; - Good biocompatibility.	- Oxidative stress; - Toxicity at high concentrations.
Hybrid	Hybrid NPs [70,71]	- Synergistic effect; - Flexibility in design and functionality.	- Complex formulation; - Stability and scalability issues.

AuNPs: gold NPs; NEM: nanoemulsions; NPs: nanoparticles; PLGA: polylactic-co-glycolic acid; SLNs: solid lipid NPs.

**Table 3:** Selected clinical trials of nanoparticle-based TB vaccines.

Vaccine	Nanoparticle Type	Antigen	Trial	Key Findings
RUTI [149,150]	Lipid-based NPs	Heat-inactivated Mtb fragments	Phase II	- Strong immune response; - Good safety profile; - Potential to reduce TB reactivation risk.
ID93 + GLA-SE [149,151,152]	Glucopyranosyl lipid A (GLA-SE)	ID93 (fusion of four Mtb antigens)	Phase I/II	- Robust Th1 response; - Improved antigen stability and immunogenicity; - Safe with manageable adverse effects.
H56:IC31 [153,154]	Peptide NPs with adjuvant	H56 (ESAT-6, Ag85B, Rv2660c)	Phase I/II	- Robust Th1-biased immunity; - Potential as a booster vaccine or adjunct to TB treatment.
M72/AS01E [155,156,157]	Liposomal NPs	M72 (fusion of Rv1196 and Rv0125)	Phase IIb/III	- ~50% efficacy in preventing active TB (adults); - Strong humoral and cellular immune responses.

Mtb: Mycobacterium tuberculosis; NPs: nanoparticles.

## Data Availability

No new data were created or analyzed in this study. Data sharing is not applicable to this article.
